# Autochthonous transmission patterns of dengue virus serotype 2 in Italy: evidence from outbreaks in 2024

**DOI:** 10.2807/1560-7917.ES.2026.31.27.2600035

**Published:** 2026-07-09

**Authors:** Carla Molina Grané, Martina Del Manso, Mattia Manica, Chiara Sacco, Francesco Menegale, Antonino Bella, Flavia Riccardo, Augusto Liverani, Alessia Pesaresi, Giovanna Mattei, Giulio Matteo, Adriano Murgano, Dalia Palmieri, Barbara Rita Porchia, Chiara Staderini, Dengue risk assessment working group, Patrizio Pezzotti, Stefano Merler, Piero Poletti, Dengue risk assessment working group, Tiziana Lazzarotto, Alessandra Fantuzzi, Giacomo Creola, Ada Mammarella, Matteo Consorte, Claudio Turchi, Flavio Valerio, Vincenzo Cordella, Sara Brugnoli, Giulia Di Pisa, Fabio Filippetti, Lolita Sebastianelli, Irene Mercuri, Francesca Diotallevi

**Affiliations:** 1 Center for Health Emergencies, Fondazione Bruno Kessler, Trento, Italy; 2 Department of Infectious Diseases, Istituto Superiore di Sanità, Rome, Italy; 3 External Relations Office and Centre for International Affairs, Istituto Superiore di Sanità, Rome, Italy; 4 Pesaro Urbino Local Health Unit, Pesaro and Urbino, Pesaro, Italy; 5 Emilia-Romagna Region Collective Prevention and Public Health Department, Bologna, Italy; 6 Prevention, Food Safety and Veterinary Service, Abruzzo Region Health Department, Italy; 7 Prevention, workplace safety and public health of Toscana Region, Italy; 8 Prevention department, Public Health Unit, Toscana Centro, Florence, Italy; 9 The members of this group are listed under Collaborators

**Keywords:** dengue, autochthonous, outbreak, Europe, Italy, transmission, transmission chain reconstruction, model

## Abstract

**BACKGROUND:**

In 2024, multiple transmission foci of dengue virus serotype 2 (DENV-2) were detected across four Italian regions, resulting in the largest number of autochthonous cases ever recorded in mainland Europe.

**AIM:**

We aimed to characterise DENV-2 transmission patterns in Italy in 2024.

**METHODS:**

We analysed 296 locally acquired cases with symptom onset between 31 July and 31 October. Using detailed spatiotemporal data, we reconstructed transmission chains between cases with well-documented exposure sites, applying a Bayesian framework. We estimated the generation time and net reproduction number (R_t_) for the main transmission foci, quantified the proportion of transmission occurred across varying distances and assessed the influence of different factors, including temperature and control interventions, on secondary transmission.

**RESULTS:**

Three major foci were identified, with peak R_t_ estimates ranging from 1.35 to 3.33. The mean generation time was 18.0 days (95% credible interval (CrI): 15.5–20.0 days). Household transmission accounted for 15.4% (95% CrI: 12.6–17.4%) of infection events. Among cases with an identified source of infection, < 1% of transmissions occurred beyond 400 m. Transmissibility declined significantly after outbreak detection, with the average number of secondary cases per infection dropping from 1.4 to 0.4. Vector control interventions were associated with a 41.0% reduction (95% CI: 6.6–63.3%) in transmission; transmission also increased by 19.8% (95% CI: 11.3–29.0%) for each 1°C rise in temperature.

**CONCLUSION:**

Autochthonous dengue outbreaks in Italy in 2024 were primarily driven by short-distance transmission. Our findings support that early case detection and rapid vector control are instrumental in reducing transmission.

**Key public health message**

**What did you want to address in this study and why?**

Dengue is a viral infection spread from mosquitoes to humans and usually found in tropical regions, such as Latin America and the Caribbean, and South-eastern Asia. In 2024, Italy experienced the largest dengue outbreak ever recorded in mainland Europe, with nearly 300 locally acquired cases, which gave us an unprecedented opportunity to better understand how the virus can spread in Europe.

**What have we learnt from this study?**

By studying where and when infections occurred, we found that most transmission happened within a few hundred metres, indicating highly localised spread. After the outbreak was detected, a combination of falling temperatures, measures aimed at reducing mosquito abundance and increased awareness of disease risk among the affected population led to a marked decline in transmission.

**What are the implications of your findings for public health?**

Timely identification of cases, combined with mosquito control interventions around detected infections, can markedly limit transmission. However, when case detection is delayed, broader control efforts may be required. Strengthening surveillance and preparedness will become increasingly important as climate conditions favour longer mosquito seasons and higher transmission risks in Europe.

## Introduction

Dengue, caused by dengue virus (DENV), is a mosquito-borne disease posing considerable public health concern due to its growing global burden and geographical expansion of affected areas [1]. Although traditionally endemic in tropical and subtropical regions [2], autochthonous infections with DENV are now increasingly reported in temperate areas of Europe, particularly in France, Italy and Spain [3-7]. In these countries, local transmission is sustained by the presence of *Aedes albopictus*, a mosquito species now firmly established in southern Europe and one of the vectors of DENV [8,9].

Autochthonous DENV transmission has been sporadically documented in mainland Europe since 2010, when 10 locally acquired cases were reported in Croatia and two in France [10,11]. Up to 2024, local transmission events remained limited in size, typically resulting in small, localised clusters of cases [3,12,13], with the two largest outbreaks involving 34 cases in France in 2022 and 45 in Italy in 2023 [4,5].

However, 2024 marked a turning point, potentially indicating Europe’s transition from sporadic *Aedes*-borne disease outbreaks to more regular seasonal transmission patterns and greater risks of larger epidemics [14]. While transmission was again observed in France and Spain, Italy alone reported 296 autochthonous cases caused by dengue virus serotype 2 (DENV-2), nearly surpassing the total number of all DENV cases recorded in mainland Europe in all previous years combined [3,7]. Of these, 226 occurred in the Marche region – most of them in the municipality of Fano – making this the largest autochthonous dengue outbreak recorded in mainland Europe [5,6,10]. Additional transmission foci of DENV-2 were identified in three other Italian regions (44 cases in Emilia-Romagna, 22 in Abruzzo and 4 in Tuscany), with preliminary evidence suggesting that different foci might have been epidemiologically linked.

In this study, we analysed detailed records of all autochthonous DENV-2 cases in Italy in 2024. By probabilistically reconstructing transmission chains among ascertained cases, we derived multiple metrics characterising DENV transmissibility over time and space, while examining individual heterogeneity in onward transmission and assessing how eco-climatic factors and control interventions influenced the likelihood of disease spread. This analysis aims to fill an existing knowledge gap on dengue transmission dynamics in temperate Europe, where understanding has been hindered by the sporadic occurrence of autochthonous cases and the fragmentary nature of available data [5].

## Methods

### Surveillance and control of dengue virus in Italy

In Italy, surveillance of DENV infections in humans is coordinated by the Ministry of Health and supported by the technical expertise of the Italian National Institute of Health (ISS), in accordance with the National Arbovirus Response Plan 2020–2025 [15]. Detection of DENV cases is performed by general practitioners or hospital physicians who request laboratory analysis by PCR or serological testing for suspected cases. Case definitions are in agreement with the European Union (EU) case definitions [16] (Box). Any probable or confirmed case identified by clinicians in a medical facility should be notified within 12 h to regional health authorities.

Box **Definition of suspected, probable and confirmed cases of dengue, Italy**

**Suspected case:**

• Any person with symptoms consistent with dengue, such as fever > 39°C, nausea or vomiting, rash, aches and pains, retro-ocular pain.

**Probable case:**

• Compatible clinical symptoms

AND

• Detection of dengue-specific IgM antibodies.

**Confirmed case:**

• Compatible clinical symptoms

AND

• Isolation of dengue virus

OR

• Detection of viral RNA

OR

• Detection of dengue viral antigen (NS1)

OR

• Detection of dengue-specific IgM antibodies in a single serum sample and confirmation by neutralisation or seroconversion or fourfold antibody titre increase in paired serum samples.

In the absence of evidence of chikungunya virus (CHIKV) or DENV infections in humans, actions to reduce man-made *Aedes* breeding sites and limit mosquito abundance are based on local (sometimes even private) decisions not coordinated or reported at the national level. Upon notification of a human case, vector control interventions, coordinated by the local health authorities, are mandatory and implemented within 24 h. Vector control focuses on insecticide disinfestation of affected areas, defined as a 200 m radius around their residence and presumed sites of exposure, with priority given to adulticide treatments in both public and private spaces. Intervention measures include identification and elimination of peri-domestic larval breeding sites through door-to-door inspections and targeted risk communication. Additional response activities may include local suspension or deferral of blood donations and screening campaigns aimed at uncovering cryptic transmission [5,6]. More details on the surveillance and control protocols in Italy can be found in Supplementary Material section 1.

### Investigation of the 2024 outbreak and mosquito control interventions

For each case, data collected by the competent local health authorities included sex, age, date of symptom onset, date of reporting, date of diagnosis, and the geographical location of residence and likely exposure. The presumed exposure location was determined through standard epidemiological investigation: patients were interviewed about all places visited during the presumed exposure window, with particular emphasis on locations frequented at dawn or dusk. This information was then integrated with entomological findings and with potential exposure sites of other probable and confirmed cases to infer for each case their most likely site of exposure. Information was also gathered on whether cases were cohabitants and on their travel history to endemic countries or to any location in Italy where at least one case had been reported. Case interviews were conducted for both probable and confirmed cases as part of the epidemiological investigation and active surveillance activities, based on protocols and questionnaires defined by local health authorities. Active case finding was carried out through testing of cohabitants and close family members of confirmed cases. Detailed records of the control interventions applied in each affected municipality were also compiled.

In 2024, most reported cases were identified in the municipalities of Fano (Marche), Cavezzo (Emilia-Romagna) and Ortona (Abruzzo). In addition to mandatory vector control interventions after the identification of each case, adulticide treatments in Fano targeting the entire municipality and up to 2 km beyond residential areas were eventually planned for 15–18 September given the high number of cases detected among residents (19 cases reported before 15 September). Due to adverse weather conditions on 17–18 September, the treatment was carried out on 15–16 and 22–23 September. Municipal-level treatments were again conducted on 6–8 October in Fano. In Cavezzo, mosquito control interventions were extended from 23 to 30 September to all public areas considered at risk, including door-to-door inspections on 24–25 September. In Ortona, the radius of interventions around a case’s residence was extended to 400 m from 4 October; interventions covered the entire city centre on 10 and 12–13 October when new cases continued to be reported, and expanded to peripheral residential areas from 12 to 17 October and from 24 to 27 October after the detection of new infections in these areas.

### Transmission chain reconstruction

We applied a Bayesian model to reconstruct likely chains of transmission in the towns reporting more than one autochthonous case, building on previously established approaches [5,17-19]. The model leverages spatiotemporal information from ascertained cases to probabilistically infer the most likely source of each infection, while simultaneously estimating key quantitative metrics that describe the transmission dynamics during the observed outbreaks.

In the context of arboviral infections, transmission between human cases occurs via a competent mosquito vector. The model estimates the likelihood that each case was caused by transmission originating from any other case, using a force of infection that depends on both the time of symptom onset of potential human infectors and the spatial coordinates of their presumed exposure location. To account for spatial heterogeneity, we used an exponentially distributed kernel to model transmission distances between cases exposed at different locations within the same town; a separate force of infection was applied for cases residing in the same household. To incorporate the possibility of unobserved or untraced infections and of misclassification of the site of exposure of cases, the model allows for each case to have been infected by an unknown source in the community, with a probability proportional to the overall number of cases in the province of residence. This accounts for infections that may have occurred through mosquito bites in locations other than those identified through case interviews. The generation time (i.e. the time elapsed between the infection of a primary case and of its secondary cases) was assumed to be gamma distributed.

Individuals for whom a plausible site of exposure could not be determined during outbreak investigations were excluded from the reconstruction of transmission chains. However, these cases were included in estimating the community-level force of infection. Data augmentation techniques were employed to incorporate uncertainty in the unobserved infection dates.

The transmission chain reconstruction allowed us to estimate the generation time distribution for DENV-2, the spatial profile of transmission distances, and the distribution of secondary cases generated by each confirmed infection. A consensus transmission chain was also derived by considering the most frequently occurring transmission links across all plausible chains identified through Markov Chain Monte Carlo (MCMC) sampling. Further details on the definition of the model and underlying assumptions are provided in the Supplementary Material section 7.

### Analysis of transmission patterns of dengue virus serotype 2

We leveraged the estimated generation time to compute the net reproduction number (R_t_) for the main transmission foci, by applying the renewal equation to the time series of symptomatic cases by date of symptom onset, using a Markov Chain Monte Carlo (MCMC) approach [5,6,20]. We also analysed individual and spatial heterogeneity in transmission based on the inferred links identified through the reconstruction of transmission chains.

We used a negative binomial generalised linear regression model (NB-GLM) with log link to investigate the risk factors associated with the number of secondary cases generated by each infected case. We considered the number of secondary infections estimated for each infected case according to the consensus chain as the response variable. Considered covariates included the age and sex of each infected case, the population density (resolution: 100 m^2^) [21] and the normalised difference vegetation index (NDVI; resolution: 300 m^2^) [22] at their location of likely exposure, and a dichotomous variable (pre-post) identifying their timing of symptom onset relative to outbreak detection (i.e. the time of the first notification of a locally acquired case within each transmission focus). Since the latter implicitly captures temporal variations in temperature, reporting practices, and the implementation of vector control measures, we further investigated the role of these factors by fitting a second NB-GLM replacing the pre-post variable with: (i) the mean temperature over a period starting from the infection date and lasting the average generation time [23-26]; (ii) a binary variable indicating whether vector control interventions were already implemented before the case symptom onset in their likely site of exposure and (iii) the time required for their reporting after symptom onset. The province of residence was included as either a fixed or random effect for sensitivity analysis. Regression analyses were carried out using R (R Project for Statistical Computing, software version 4.4.2, https://www.r-project.org/). Further details on temperature data and reproduction number and regression analyses are provided in the Supplementary Material sections 5, 8 and 9, respectively.

## Results

### Epidemiological investigations

In Italy, the first case of DENV-2 infection in 2024 was diagnosed on 5 September in the town of Ortona, located in the province of Chieti (Abruzzo region), although immediate epidemiological investigations could not identify any evidence of local transmission. On 11 September, the second case of DENV-2 infection was diagnosed in the town of Fano (ca 200 km from Ortona), in the province of Pesaro and Urbino (Marche region). Shortly after, epidemiological investigations revealed sustained local transmission in this area and pointed to a secondary cluster of cases in the hamlet of Bellocchi, situated within the municipality of Fano, along with scattered cases across the same province [6]. On 13 September, an isolated case was also identified in the province of Siena (Tuscany region). On 17 September, further clusters of autochthonous cases were identified in Cavezzo, in the province of Modena (Emilia-Romagna region), and on 26 September in the province of Chieti (mainly in Ortona). Finally, on 7 October, a family cluster was identified in a town in the province of Florence (Tuscany), and one isolated case was identified on 31 October in the province of Ancona (Marche region). None of the identified cases reported any history of international travel. The geographical distribution of the cases within Italy is available in Supplementary Figure S2.

In total, throughout 2024, 296 probable and confirmed autochthonous DENV-2 infections were identified in Italy, with symptom onset dates ranging from 31 July to 31 October 2024. The time series of cases by province is available in Supplementary Figure S3. Overall, the mean reporting delay, defined as the time between symptom onset and the date of case reporting to the regional health authorities, was 18.2 days (95% quantile interval: 3.0–60.6 days), reflecting delays in care-seeking, clinical identification and investigation of suspected cases. When stratified by the timing of symptom onset relative to the date of local transmission detection, the mean reporting delay was 28.3 days (95% quantile interval: 3.9–70.0 days) for cases occurring before outbreak identification and 11.5 days (95% quantile interval: 2.4–34.9 days) for cases occurring afterwards, when awareness of local transmission had substantially increased among both the general public and clinicians. Meanwhile, as part of entomological surveillance, DENV-2 was detected in *Ae. albopictus* mosquito pools in Fano on 16 and 21 September, in Cavezzo on 20 and 25 September, and in Ortona between 20 September and 1 October.

Of the 296 autochthonous cases, 286 were reported among residents of three municipalities: Fano (222 cases, including 15 in the hamlet of Bellocchi), Cavezzo (43 cases) and Ortona (21 cases). The median age of cases was 63 years (IQR: 44–74 years), with a male-to-female ratio of 1.04 (males: 51.0%) (Table).

**Table:** Description of the identified autochthonous cases with dengue virus serotype 2 infection, Italy, 2024 (n = 296)

Characteristic	Cases	
	n	%
A. Region		
Marche region (Pesaro and Urbino and Ancona provinces)		
Fano municipality		
Fano town	199	67.2
Bellocchi^a^	13	4.4
Unknown site of exposure	10	3.4
Other municipalities		
Known site of exposure	2	0.7
Unknown site of exposure	2	0.7
Emilia-Romagna region (Modena province)		
Cavezzo municipality		
Known site of exposure	40	13.5
Unknown site of exposure	3	1.0
Other municipalities		
Unknown site of exposure	1	0.3
Abruzzo region (Chieti province)		
Ortona municipality		
Known site of exposure	13	4.4
Unknown site of exposure	8	2.7
Other municipalities		
Unknown site of exposure	1	0.3
Tuscany region (Florence and Siena provinces)		
Known site of exposure	3	1.0
Unknown site of exposure	1	0.3
B. Sex		
Male	151	51.0
Female	145	49.0
C. Age (years)		
0–19	14	4.7
20–49	78	26.4
50–69	102	34.5
≥ 70	102	34.5

^a^ The likely site of exposure was known for 13 out of 15 residents of Bellocchi.

More than half (n = 168) of all cases were aged ≥ 60 years. Individuals aged ≥ 60 years appeared to be overrepresented among cases compared with the underlying population of the affected municipalities [27], whereas younger individuals were often underrepresented. Further details on the age distribution of cases are available in Supplementary Material section 6. All ascertained infections were symptomatic.

Despite extensive investigations, the primary case initiating local transmission – whether travel-related or acting as a link between different locations and seeding secondary transmission foci – could not be identified in any of the clusters. However, a family of three from a town in the province of Florence travelled to Fano in August. Two members spent a week in central Fano (23–31 August) and developed symptoms on 8 September; the third visited Fano on 31 August and developed symptoms on 19 September. Additionally, one case residing in Cavezzo, who developed symptoms on 12 August, spent two non-consecutive nights in a small village located ca 5 km from Fano on 25–26 July and 29–30 July. Finally, the first DENV-2 case residing in Ortona, who developed symptoms on 18 August, spent two nights (14–16 August) in a village in the province of Rimini (Emilia-Romagna), ca 30–40 km from Fano and ca 190 km from Cavezzo, where no further cases were identified.

Epidemiological interviews conducted during the outbreak investigations identified 270 cases whose probable exposure occurred near other notified cases. In contrast, the place of exposure could not be reliably determined for 26 cases (8.8%). Although we did not perform probabilistic inference of likely infectors for these cases, their contribution was fully incorporated into the estimation of infection risk for other individuals.

### Generation time and temporal changes in the transmissibility of dengue virus serotype 2

**Figure 1:**
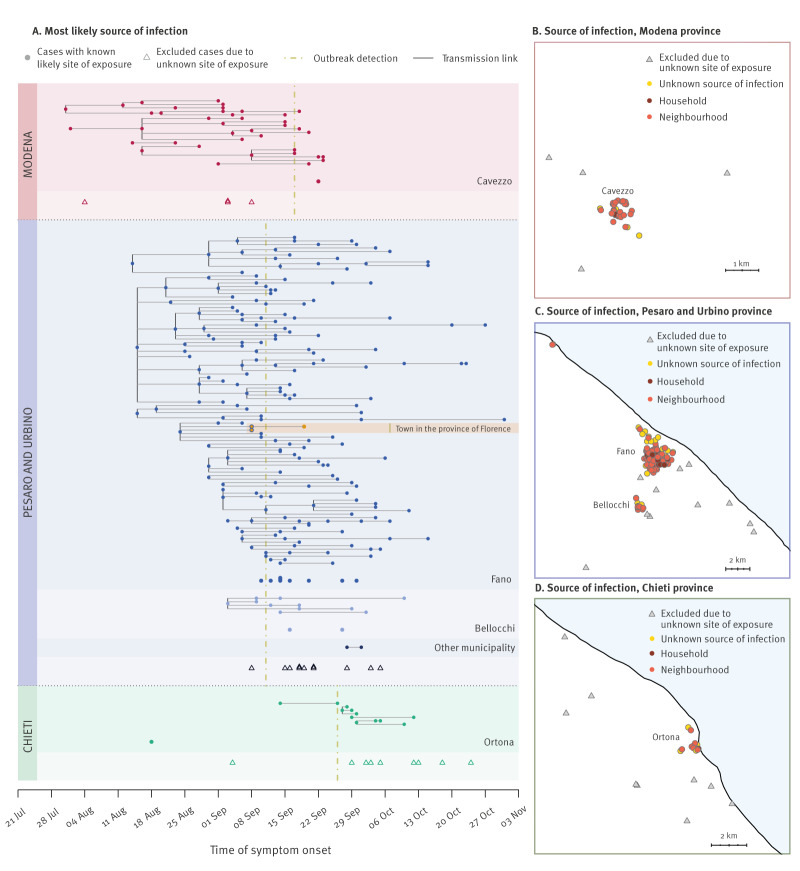
Transmission chains and geolocation of cases with dengue virus serotype 2 in the provinces of Modena, Pesaro and Urbino, Florence and Chieti, Italy, 2024 (n = 294)^a^

From the analysis of the most likely sources of infection associated with 270 ascertained cases (Figure 1), the DENV-2 generation time was estimated to follow a gamma distribution, with a mean of 18.0 days (95% credible interval (CrI): 15.5–20.0 days) and a standard deviation of 10.3 days (95% CrI: 8.3–11.2 days). This indicates that ca 50% of secondary infections attributable to a specific human case occurred between 10 and 23 days after the source case’s time of infection.

DENV-2: dengue virus serotype 2.

^a^ Provinces of Modena, Pesaro and Urbino, Florence and Chieti reported 294 cases; two additional cases, not included in the figure, were identified in Ancona and Siena provinces.

Panel A. Consensus transmission chains resulting from the probabilistic model inferring the most likely location of exposure for the 270 cases reported across different transmission foci (province of Modena (town of Cavezzo) in red; province of Pesaro and Urbino (towns of Fano, Bellocchi, and other) in blue; province of Chieti (town of Ortona) in green; cases from the town in the province of Florence linked with the cluster of Fano are highlighted in orange). Cases identified in the main foci with plausible location of likely exposure are marked with a circle; isolated cases identified in surrounding areas within the same province with unknown exposure site are marked with a triangle. Cases are displayed by day of symptom onset, and the time of detection of each transmission foci is marked with a dashed yellow line.

Panels B, C, D. The maps show the geolocation of all DENV-2 cases reported in each of the main affected provinces, with colours identifying their source of infection according to the consensus chain. Red and orange circles present cases for which a specific individual was identified as their most likely infector: in red, individuals who likely acquired the infection in their household. Yellow circles present cases for which a specific infector was not assigned. Grey triangles present the residences of cases for which no plausible place of likely exposure was identified, and who were thus excluded from the transmission chain reconstruction.

**Figure 2:**
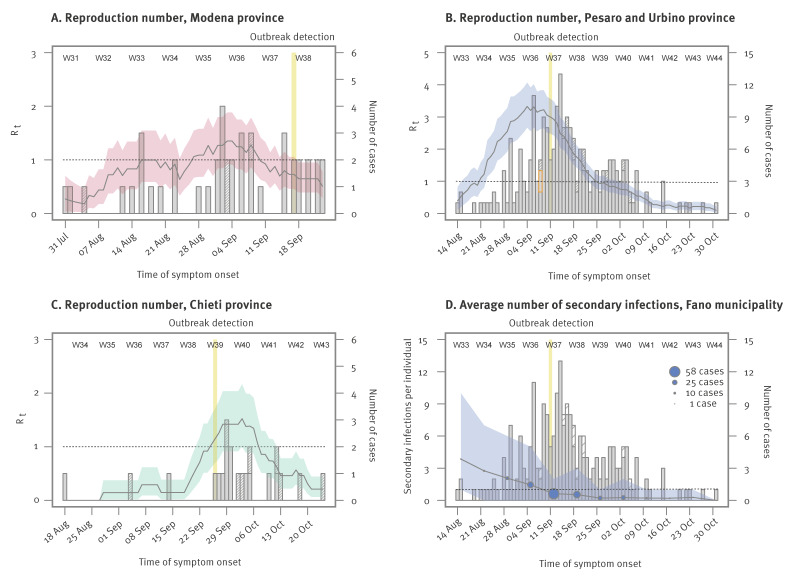
Estimated daily net reproduction number (R_t_) (Panels A–C, n = 293)^a^ and average number of secondary infections per individual per week in Fano municipality (Panel D, n = 222) of dengue virus serotype 2, Italy, 2024

Leveraging the estimated generation time, we separately computed R_t_ from the time series of all cases reported in the provinces of Pesaro and Urbino, Chieti and Modena. We found that R_t_ peaked at 3.33 (95% CrI: 2.67–4.07), 1.52 (95% CrI: 1.01–2.16) and 1.35 (95% CrI: 0.91–1.88) in the three provinces, respectively (Figure 2). In the first two provinces (Pesaro and Urbino and Chieti), R_t_ dropped below the epidemic threshold of 1 approximately 2 weeks after the outbreak was detected (i.e. following the first notification of a locally acquired case), which prompted enhanced surveillance and targeted vector control measures. In Modena province, R_t_ hovered around this threshold during the initial epidemic phase and declined shortly after the detection of local autochthonous cases.

Crl: credible interval; R_t_: net reproduction number; W: week.

^a^ Case numbers: Modena (n = 44 cases), Chieti (n = 22 cases) and Pesaro and Urbino (n = 227 cases), with two of them residents in a town in the province of Florence but infected in Fano.

Panels A, B, C. Solid coloured lines present mean estimates; shaded areas present 95% CrI. Solid grey bars present cases with known site of exposure, and dashed bars cases with unknown site of exposure; together they present the time series associated with the estimated transmissibility.

Panel B. The orange rectangle marks two cases residing in a town in the province of Florence, but who were likely infected in Fano (exported cases).

Panel D. The average number of secondary infections per individual each week in Fano is depicted in blue, as obtained by pooling together all plausible transmission chains. Solid grey bars present cases with known site of exposure included in the transmission reconstruction model; dashed grey bars are cases with unknown site of exposure. Points’ size is proportional to the number of cases with symptom onset in that week with known exposure site, presenting the underlying sample size associated with the corresponding estimate. Blue line with points presents mean estimates; shaded area presents 95% CrI.

In all panels, the timing of outbreak detection is indicated in yellow.

Pooling all plausible transmission chains compatible with the spatiotemporal distribution of cases observed in the Fano outbreak, we derived an alternative metric of transmissibility over time, based on the reconstructed number of secondary infections generated by each reported case, by date of symptom onset (Figure 2D). Our findings suggest that symptomatic cases initially generated, on average, more than one secondary infection. However, this value declined below the epidemic threshold of one shortly after outbreak detection.

**Figure 3:**
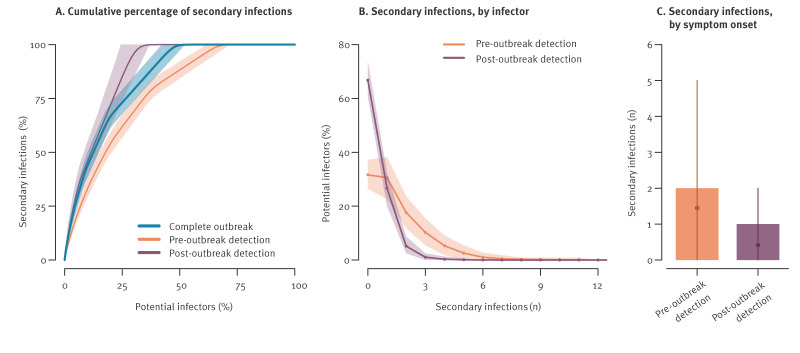
Cumulative percentage of secondary infections (Panel A), number of secondary infections by infector (Panel B) and distribution of secondary infections by symptom onset (Panel C) of dengue virus serotype 2, Italy, 2024 (n = 270)

Consistently, when considering all identified transmission foci, individuals with symptom onset after outbreak detection exhibited decreased average transmissibility compared with earlier cases, generating on average 0.4 (95% CrI: 0–2) secondary cases compared with a mean of 1.4 (95% CrI: 0–5) before detection (Figure 3C).

Crl: credible interval; DENV-2: dengue virus serotype 2.

Panel A. Cumulative percentage of secondary infections caused by DENV-2 cases, with potential infectors ordered from those who infected the largest number of cases to those who did not infect any secondary case. Estimates are shown for all individuals with a plausible exposure site (blue), and separately for cases with symptom onset before (orange) or after (purple) outbreak detection. Solid lines represent mean model estimates; shaded areas represent 95% CrI.

Panel B. Density distribution of the number of secondary cases caused by each potential infector with symptom onset before (orange) and after (purple) outbreak detection. Solid lines represent mean model estimates; shaded areas represent 95% CrI.

Panel C. Distribution of secondary infections when stratifying cases by symptom onset before (orange) or after (purple) outbreak detection. Points indicate mean estimates; boxes represent the 50% CrI; vertical lines denote the 95% CrI.

### Individual heterogeneity in the offspring distribution and distance of transmission

Beyond average trends, the number of secondary infections exhibited substantial heterogeneity at the individual level. This was particularly evident among individuals with symptom onset after outbreak detection, where 20% of infected individuals were responsible for 72.2% (95% CrI: 62.5–85.7%) of ascertained cases. In this group, 33.2% (95% CrI: 26.3–39.4%) generated at least one secondary case, and only 6.5% (95% CrI: 3.8–10.0%) caused two or more cases (Figure 3). By contrast, among individuals with symptom onset before the outbreak detection, 68.4% (95% CrI: 62.7–73.6%) generated at least one secondary case, and 9.9% (95% CrI: 5.5–14.5%) caused four or more.

We estimated that 15.4% (95% CrI: 12.6–17.4%) of cases could be traced back to an infectious household member, while 68.3% (95% CrI: 63.3–76.3%) were linked to a specific case infected close to their likely exposure site. For the remaining 16.3% (95% CrI: 7.4–21.5%), the infection was likely caused by viral circulation within one of the main transmission foci but could not be attributed to a specific individual case (Figure 1). Based on the DENV incubation period, ranging from 3 to 10 days, any epidemiological link between the diagnosed cases in Fano and the case residing in Cavezzo who visited the surroundings of Fano was considered highly unlikely. Conversely, at least one of the three cases in the family cluster recorded in a town in the province of Florence (Tuscany) was likely infected in Fano, and the last case in the family was likely infected within the household. Due to the overlapping exposure period in Fano and the identical symptom onset of the first two cases, it was not possible to determine which of the two was the index case in the household, nor to exclude household transmission between them.

**Figure 4:**
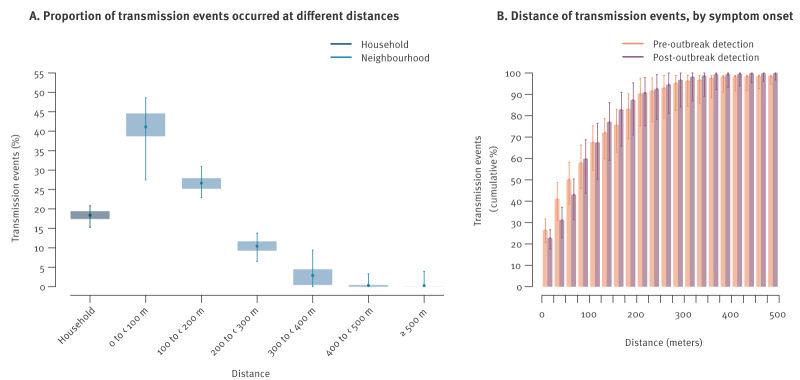
Estimated distances of transmission events of dengue virus serotype 2, Italy, 2024 (n = 270)^a^

More in general, we found that transmission was predominantly focal. Among individuals for whom a likely source of infection could be identified (on average, 83.7% of analysed cases), over half of transmission events occurred < 100 m of each other’s probable exposure sites: 18.4% (95% CrI: 15.3–20.8%) within the same household and 41.1% (95% CrI: 27.5–48.6%) within the immediate neighbourhood where the primary case was likely exposed (Figure 4A). The likelihood of transmission decreased with increasing spatial separation: 26.6% (95% CrI: 22.9–30.9%) of events were associated with exposure sites located from 100 to < 200 m apart and 10.4% (95% CrI: 6.5–13.8%) with sites from 200 to < 300 m apart. Transmission beyond 400 m was rare, accounting for less than 1.0% (mean: 0.59, 95% CrI: 0–7.2%) of identified links.

Crl: credible interval.

^a^ Estimates account for individuals for whom a likely source of infection could be identified (83.7% on average).

Panel A. Distribution of the transmission distance among individuals for whom a likely source of infection could be identified, as estimated from the reconstructed transmission chains. Points indicate mean model estimates; boxes present 50% CrI; whiskers represent 95% CrI.

Panel B. Cumulative percentage distribution of the transmission distance, distinguishing between cases with symptom onset before (orange) and after (purple) outbreak detection. Rectangles indicate mean model estimates; whiskers represent 95% CrI.

When comparing transmission distances from likely sources of infection for cases with symptom onset before and after outbreak detection, a lower proportion of transmission occurred within a short spatial range after detection, in line with mosquito control measures mandated within a 200 m radius centred on the likely exposure site of each case (Figure 4B).

### Key factors influencing secondary transmission events

Regression model results showed that cases with symptom onset before outbreak detection were associated with approximately four times (mean: 3.7, 95% confidence interval (CI): 2.7–5.2) more secondary infections, and that males were associated with 41.3% (95% CI: 4.4–91.7%) more secondary cases than females. By further analysing key factors that might have played a role in changing the individuals’ temporal transmissibility, we found that the number of secondary cases was (i) reduced by 41.0% (95% CI: 6.6–63.3%) when vector control interventions were carried out in the likely exposure site of the potential infector before their symptom onset, and (ii) increased by 19.8% (95% CI: 11.3–29.0%) for each 1°C rise in average temperature during their post-infection period. Consistent results were obtained when including the province of residence of cases as either a fixed or random effect. Regression details and quantitative results are available in Supplementary Material section 9.

## Discussion

The DENV-2 outbreaks in Italy in 2024 may represent a major turning point, characterised by high transmissibility, multiple transmission foci across four regions and an unprecedented case count within a single season in a temperate European country. Data collected through active case surveillance enabled us to perform a comprehensive quantitative analysis of DENV transmission patterns in Italy, addressing a critical gap resulting from the sporadic occurrence of cases in non-endemic settings.

Among cases with an identified source of infection, less than 1% of secondary transmissions occurred beyond 400 m from the primary case (with ca 15% of all cases traced back to an infectious household member), indicating that DENV transmission was highly focal – consistent with the limited flight range of the mosquito vector [28]. This suggests that, in principle, control interventions around each case should be able to prevent a substantial proportion of onward transmission. However, although all identified cases were symptomatic, the delay between the first date of symptom onset and the date of first reporting in each of the three main affected provinces ranged from 22 to 48 days, potentially affecting the timely implementation and effectiveness of vector control measures. The individual onset-to-reporting delay decreased after the recognition of local dengue transmission, likely reflecting both a prompter care-seeking behaviour among individuals with compatible symptoms and an earlier consideration of dengue in the diagnostic workup of patients with no recent travel history. Additionally, for 8.8% of cases, the likely exposure site could not be determined. Combined with an estimated mean generation time of 18.0 days, these findings suggest that expanding mosquito control interventions to a broader area – potentially the entire affected town or municipality – may be necessary to timely interrupt the transmission when multiple cases are identified in a short period of time.

Our estimates of transmission distance closely align with those reported for DENV-1 during a smaller outbreak in northern Italy in 2023 [5], as well as with data from multiple DENV serotypes in endemic regions [18], and with chikungunya virus in both endemic and non-endemic settings [17,19]. Similarly, the estimated mean DENV generation time is consistent with previous observations [5,18].

Although the reproduction number during the outbreak in Fano (Pesaro and Urbino province) reached high values typical of endemic regions (R_t_ > 3 at the peak) [18,29], our results reveal considerable spatiotemporal and individual heterogeneity in DENV transmission. On average, following outbreak detection and the associated increase in public awareness and implementation of control measures, the proportion of cases contributing to onward transmission declined from ca 70% to 30%, with 20% of infected individuals responsible for more than 70% of ascertained cases.

Consistent with previous findings, we observed that after outbreak detection, the net reproduction number fell below the epidemic threshold of one within one generation time [5,6,30]. In addition, for the first time, we disentangled the relative contributions of temperature changes and control interventions in shaping disease spread. We found that the number of secondary infections resulting from a DENV case was, on average, 41% lower when interventions were applied around the likely exposure site of the case before their symptom onset. While the exact magnitude of this reduction is uncertain, the result underscores the critical role of timely interventions in reducing transmission. In parallel, the drop in autumn temperatures likely contributed to reducing transmission, as we estimated that each 1°C increase in temperature was associated with an approximate 20% rise in transmission – a concerning trend in light of climate changes, which are extending the mosquito breeding season. The higher transmissibility observed in male cases compared with females may reflect behavioural differences in daily activities or in the use of protective measures against mosquito bites, although the extent of this difference remains uncertain. The higher attack rate observed among individuals aged ≥ 60 years may be explained by multiple factors, including a potentially greater exposure risk in older adults or lower disease severity among younger individuals.

Our findings should be interpreted considering the following limitations. Vector-borne diseases are inherently complex due to the pivotal role of vectors in the transmission process, and multiple factors may act as confounders in the analysis of DENV transmission dynamics. One key challenge is the accurate identification of likely exposure sites. Specifically, for 26 of the 296 cases, it was not possible to reliably identify any plausible site of infection. Although the rate of asymptomatic infections appears to be low in non-endemic areas [5,6], asymptomatic or mildly symptomatic cases may have gone unrecognised, potentially obscuring key transmission pathways. In addition, multiple exposure sites were not considered for the same individual. This may have biased estimates of individual-level transmissibility and led to an overestimation of the generation time, although the latter is consistent with the literature [5,18]. Even though under-detection of cases may bias the identification of transmission links, the presence and temporal variation of reporting delays should not affect the reconstruction of transmission chains or the estimation of R_t_, as both rely on symptom onset dates rather than notification dates. While we confirmed a plausible transmission link between cases in the Marche and Tuscany regions, undetected links between other affected regions cannot be excluded. In this context, as no imported case associated with these outbreaks was identified, the availability of genomic data for the identified infections would allow investigation of the origin of the circulating strains in the different transmission foci, thereby enhancing the ability to confirm epidemiological links both within and between affected municipalities, clarifying whether the outbreaks resulted from single or multiple introductions into Italy, and improving our understanding of their geographic spread in the context of transmission mediated by *Ae. albopictus*. Finally, while our regression analysis aimed to identify key factors shaping disease spread, we cannot exclude the influence of factors resulting not significant or that were not included in the model. For instance, the role of reporting delay might have been potentially masked by partial collinearity with temperature and control measures. Similarly, the vector-to-host ratio is expected to play a critical role in arboviral transmission, but we were unable to incorporate explicit data on this factor at a fine spatial scale.

The strength of this study lies in the large number of cases analysed and the use of a methodology that explicitly accounts for uncertainty in reconstructing transmission chains. Rather than relying solely on epidemiological links identified through case interviews, we employed a detailed statistical model that infers the most likely infector–infectee pairs, by considering all transmission events that are consistent with the observed data. This approach also enabled the identification of the main determinants of individual-level transmissibility, while adjusting for potential confounders.

## Conclusion

Our findings indicate that dengue transmissibility in Italy can be substantial in areas where *Ae. albopictus* is established. The high proportion of focal transmission suggests that, when cases are diagnosed promptly, vector control and active case finding within a buffer of ca 400 m around detected cases may substantially reduce onward transmission. However, when delays in case detection occur, extending mosquito control to nearby surrounding areas or at the municipality level might be advisable to cope with the undetected transmission spread. Strengthening public communication and clinical awareness may enhance early detection and outbreak control during the summer months.

## Data Availability

The data contain confidential information, and public data deposition is not permitted. Due to the sensitive nature of the data, raw data can only be made available by the Istituto Superiore di Sanità (Italian National Institute of Health) through a data-sharing agreement directly with the user (contact mail: sorveglianza.arbovirosi@iss.it).

## Members of the Dengue risk assessment working group with affiliations

Microbiology Unit, CRREM, IRCCS Azienda Ospedaliero-Universitaria di Bologna and Department of Medical and Surgical Sciences, University of Bologna, Bologna: Tiziana Lazzarotto; Modena Local Health Unit, Modena: Alessandra Fantuzzi and Giacomo Creola; Hygiene, Epidemiology, and Public Health Service, ASL 2 Lanciano-Vasto Chieti, Abruzzo Region: Ada Mammarella, Matteo Consorte and Claudio Turchi; Animal Health Veterinary Service, ASL 2 Lanciano-Vasto Chieti, Abruzzo Region: Flavio Valerio; Prevention Department, Public Health Unit, Toscana Centro, Firenze: Vincenzo Cordella, Sara Brugnoli and Giulia Di Pisa; Regional Health Agency, Marche Region, Ancona: Fabio Filippetti and Lolita Sebastianelli; Pesaro Urbino Local Health Unit, Pesaro: Irene Mercuri and Francesca Diotallevi.

## Supplementary Data

7691000 Supplement
