# Supplementary material for: Autochthonous transmission patterns of dengue virus serotype 2 in Italy: evidence from outbreaks in 2024
Source: Euro Surveill. 2026 Jul 9;31(27):2600035. doi: 10.2807/1560-7917.ES.2026.31.27.2600035 (PMC13354936; doi:10.2807/1560-7917.ES.2026.31.27.2600035)
Supplement: Supplement [file 26-00035_POLETTI_Supplement.pdf]

This supplementary material is hosted by Eurosurveillance as supporting information alongside the article *Autochthonous transmission patterns of dengue virus serotype 2 in Italy: evidence from outbreaks in 2024*, on behalf of the authors, who remain responsible for the accuracy and appropriateness of the content. The same standards for ethics, copyright, attributions and permissions as for the article apply. Supplements are not edited by Eurosurveillance and the journal is not responsible for the maintenance of any links or email addresses provided therein.

Supplementary Material

Autochthonous transmission patterns of dengue virus serotype 2 in Italy: evidence from outbreaks in 2024

Carla Molina Grané[1], Martina Del Manso[2], Mattia Manica[1], Chiara Sacco[2], Francesco Menegale[1], Antonino Bella[2], Flavia Riccardo[3], Augusto Liverani[4], Alessia Pesaresi[4], Giovanna Mattei[5], Giulio Matteo[5], Adriano Murgano[6], Dalia Palmieri[6], Barbara Rita Porchia[7], Chiara Staderini[8], Dengue risk assessment working group[†], Patrizio Pezzotti[2,*], Stefano Merler[1,*], Piero Poletti[1,*].

[1] Center for Health Emergencies, Bruno Kessler Foundation, Trento, Italy
[2] Department of Infectious Diseases, Istituto Superiore di Sanità, Rome, Italy
[3] External Relations Office and Centre for International Affairs, Istituto Superiore di Sanità, Rome, Italy
[4] Pesaro Urbino Local Health Unit, Pesaro and Urbino, Pesaro, Italy
[5] Emilia-Romagna Region Collective Prevention and Public Health Department, Bologna, Italy
[6] Prevention, Food Safety and Veterinary Service, Abruzzo Region Health Department, Italy
[7] Prevention, workplace safety and public health of Toscana Region, Italy
[8] Prevention department, Public Health Unit, Toscana Centro, Florence, Italy

[*] These authors contributed equally to the work and share last authorship.
Correspondence: Piero Poletti (poletti@fbk.eu)

[†] Dengue risk assessment working group:
Tiziana Lazzarotto[A,B], Alessandra Fantuzzi[C], Giacomo Creola[C], Ada Mammarella[D], Matteo Consorte[D], Claudio Turchi[D], Flavio Valerio[E], Vincenzo Cordella[F], Sara Brugnoli[F], Giulia Di Pisa[F], Fabio Filippetti[G], Lolita Sebastianelli[G], Irene Mercuri[H], Francesca Diotallevi[H]

[A] Microbiology Unit, CRREM, IRCCS Azienda Ospedaliero-Universitaria di Bologna, Bologna, Italy
[B] Department of Medical and Surgical Sciences, University of Bologna, Bologna, Italy
[C] Modena Local Health Unit, Modena, Italy
[D] Hygiene, Epidemiology, and Public Health Service, ASL 2 Lanciano-Vasto Chieti, Abruzzo Region, Italy
[E] Animal Health Veterinary Service, ASL 2 Lanciano-Vasto Chieti, Abruzzo Region, Italy
[F] Prevention Department, Public Health Unit, Toscana Centro, Firenze, Italy
[G] Regional Health Agency, Marche Region, Ancona, Italy
[H] Pesaro Urbino Local Health Unit, Pesaro, Italy

SUPPLEMENTARY MATERIAL **1**

1. Surveillance and control of CHIKV and DENV infections in Italy 3
2. Flowchart of all dengue autochthonous cases in Italy identified in 2024 4
3. Geographic distribution of the DENV-2 autochthonous outbreaks in Italy 5
4. Time series of cases by province 6
5. Mean daily temperatures and time series of the three main foci 7
6. Age / sex distribution of the cases 8
   6.1. Age distribution of DENV-2 cases relative to the underlying population in Fano 8
7. Transmission chains reconstruction model 9
   7.1. Generation time estimates 12
   7.2. Map of the transmission chain reconstruction in Fano center 13
8. Net reproduction number 14
9. Regression analysis 15

References 23

1. Surveillance and control of CHIKV and DENV infections in Italy

The surveillance, response and control of arboviral infections in Italy is coordinated by the Ministry of Health with the technical-scientific know-how of the Italian National Institute of Health (ISS) and implemented by the regions and autonomous provinces according to the National Arbovirus Response Plan 2020-2025 (1). In the Italian regionalized health system organization, Regional local health units are in charge of reporting the occurrence and most likely place of exposure of acute human arboviral infections to the National Surveillance System and coordinate activities in the event of health emergencies. Human surveillance of imported and locally acquired cases of DENV and CHIKV is active throughout the year, with case definitions aligned with the EU case definition (2). Actions to reduce the presence of man-made *Aedes* breeding sites and limit abundance, in the absence of evidence of CHIKV/DENV human infection, are performed routinely mainly to reduce nuisance through communication campaigns and local mosquito control programs, and are neither coordinated nor reported at the national level. Any detection of *Aedes*-borne human infections in Italy (imported or local) is performed by clinicians (GPs or hospitals depending on case severity) who request laboratory confirmation (via polymerase chain reaction (PCR) or serological tests). Any probable/confirmed case as per the EU case definition (2) triggers mandatory reporting within 12 hours to the Public Health services and local response within 24 hours including case investigation, vector capture, vector control and risk communication activities. In the presence of either confirmed or probable human cases of arbovirus infection, whether imported or autochthonous, the competent health authority activates the vector control interventions within 24 hours of notification. Control interventions are based on disinfestation of the affected area (approximately 200 meters radius around the place where the human case presumably was exposed) with insecticides, giving priority to adulticide interventions, both on public land and on private premises, and research and elimination of peri-domestic larval breeding sites, with "door-to-door" inspections of the homes included in the reported area. If a locally acquired case is suspected, based on the patient interviews, local level active case investigation, proximity vector monitoring (including xenomonitoring) and control are further enhanced. This is why the presence of CHIKV and DENV also in local mosquito pools were confirmed during observed outbreaks (3). Substance of Human Origin (SoHO) safety measures (CHIKV/DENV testing/suspension) are implemented at municipality level upon confirmation of autochthonous transmission as well as a 28-day deferral from donation of people who travelled in the affected areas. Additional response measures include national and regional enhanced support for surveillance and active case finding (including voluntary screening campaigns), national referral laboratory activities (including genomic epidemiology), medical entomology and risk communication. Activities conducted by local health authorities include individual case investigations that are not reported at national level. Similarly, control activities aimed at preventing mosquito population growth in the absence of suspected cases are not reported at the national level.

2. Flowchart of all dengue autochthonous cases in Italy identified in 2024

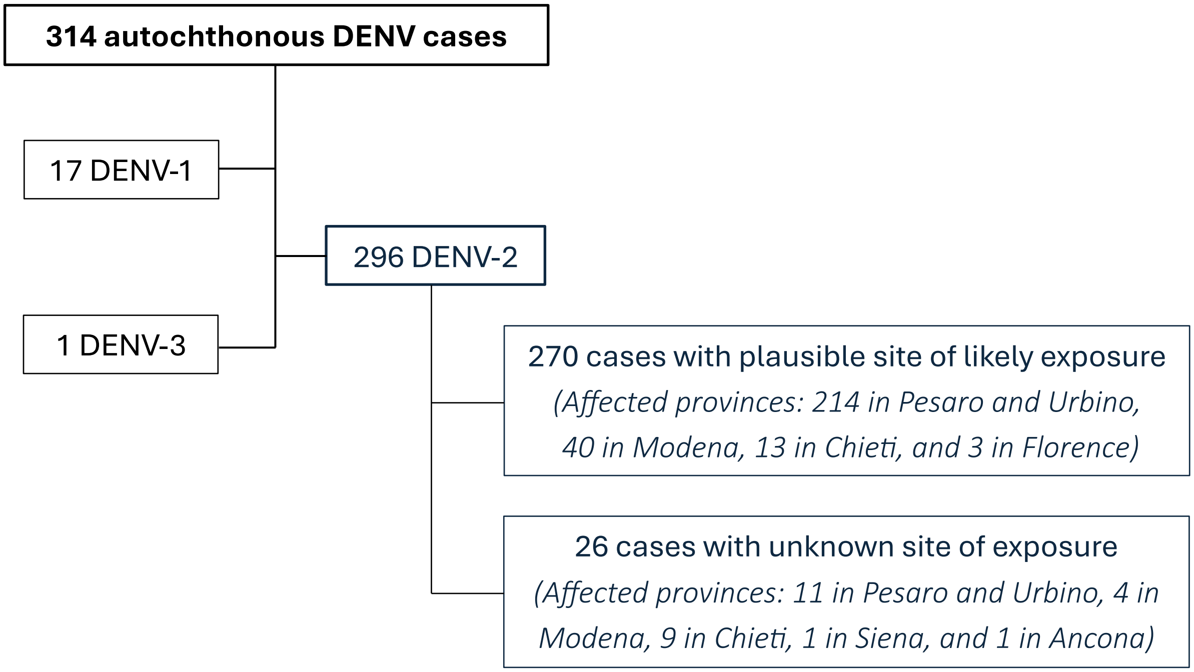

Figure S1. *Flowchart of all dengue cases—different serotypes—identified in Italy in 2024.*

3. Geographic distribution of the DENV-2 autochthonous outbreaks in Italy

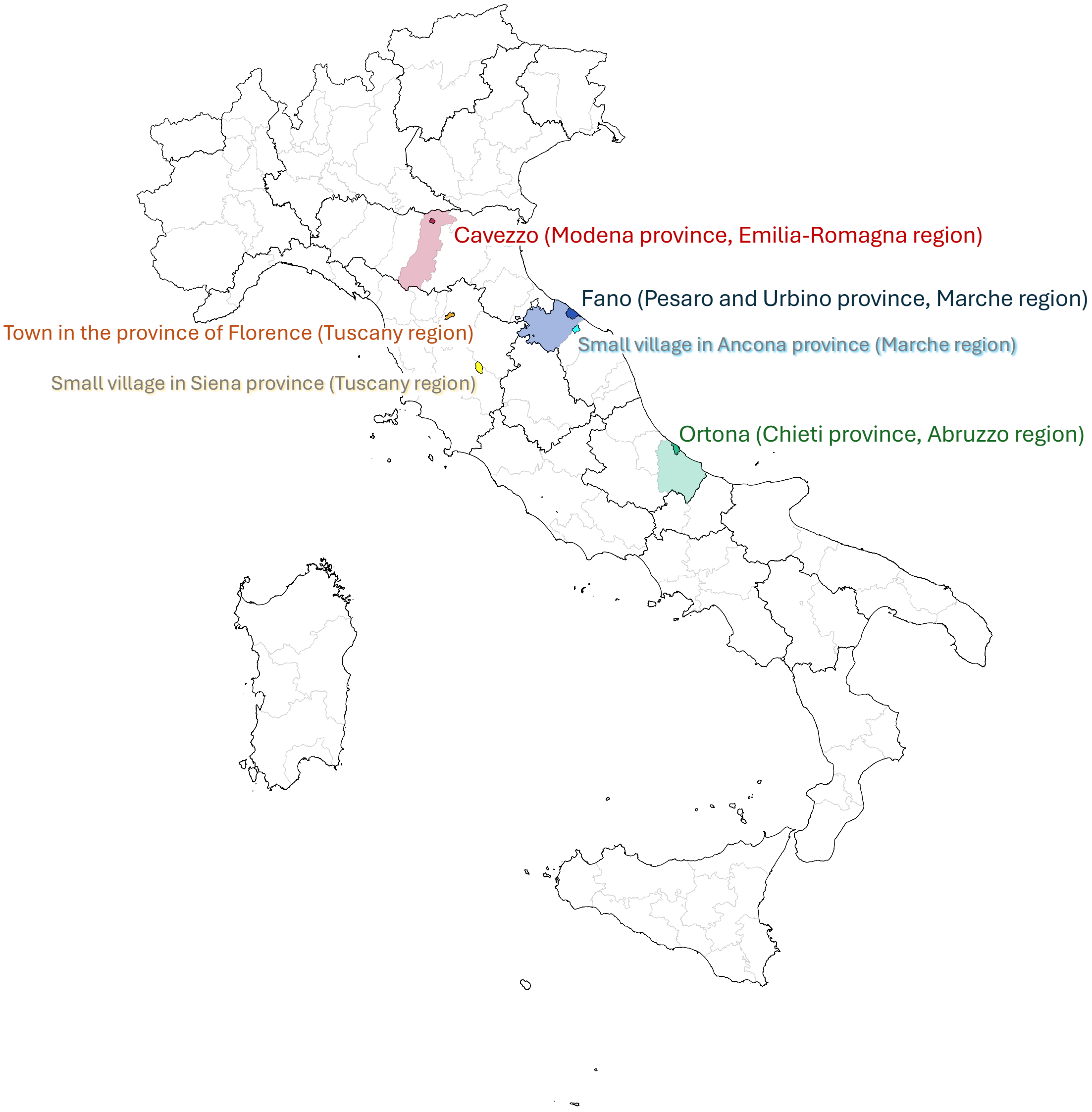

Figure S2. *Geographic distribution of the DENV-2 cases in Italy. Shaded areas represent the main affected provinces, with red, blue, and green corresponding to Modena, Pesaro and Urbino, and Chieti, respectively. The main municipalities affected within these provinces—Cavezzo, Fano, and Ortona—are highlighted. The town in the province of Florence (3 cases), the village in the province of Siena (1 case), and the village in the province of Ancona (1 case) are represented in orange, yellow, and light blue, respectively. Regional boundaries are delineated in black, and provincial boundaries in grey.*

4. Time series of cases by province

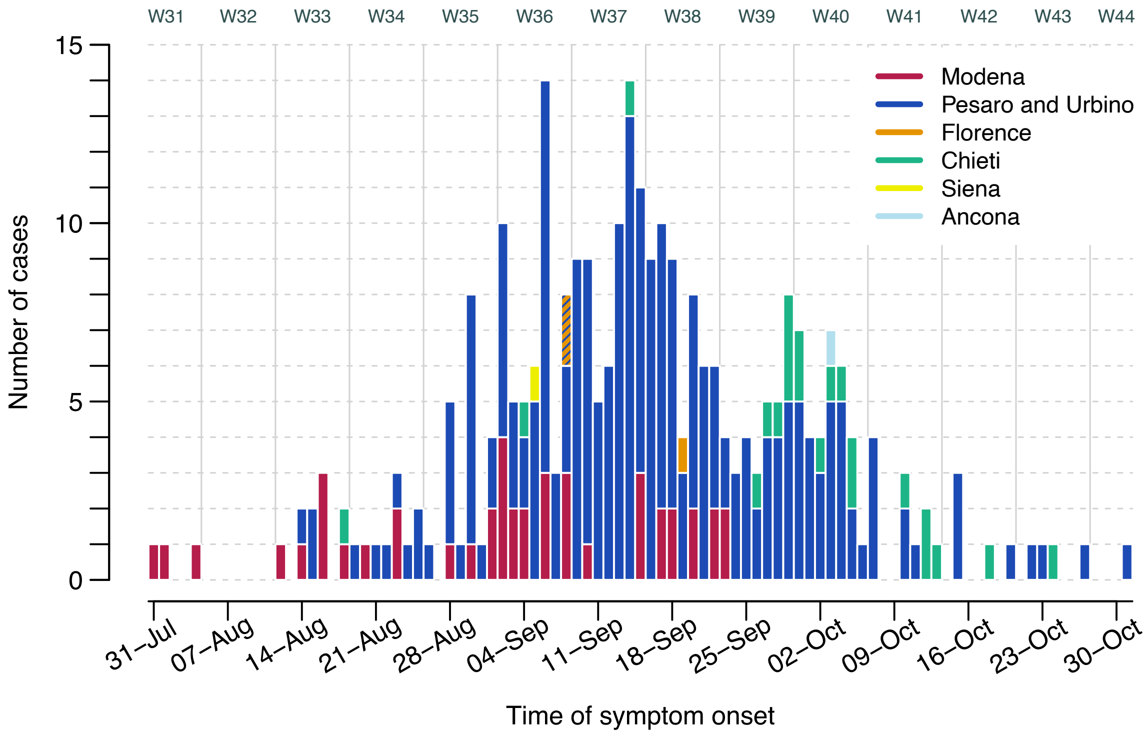

Figure S3. *Time series by date of symptom onset of all cases identified in the different provinces. In red, blue, orange, green, yellow, and light blue cases from the provinces of Modena, Pesaro and Urbino, Florence, Chieti, Siena, and Ancona, respectively. The two cases from the town in the province of Florence that spent a week in Fano while the outbreak in the region was ongoing are marked with dashed blue lines. Week numbers, at the top, according to the ISO date standard.*

5. Mean daily temperatures and time series of the three main foci

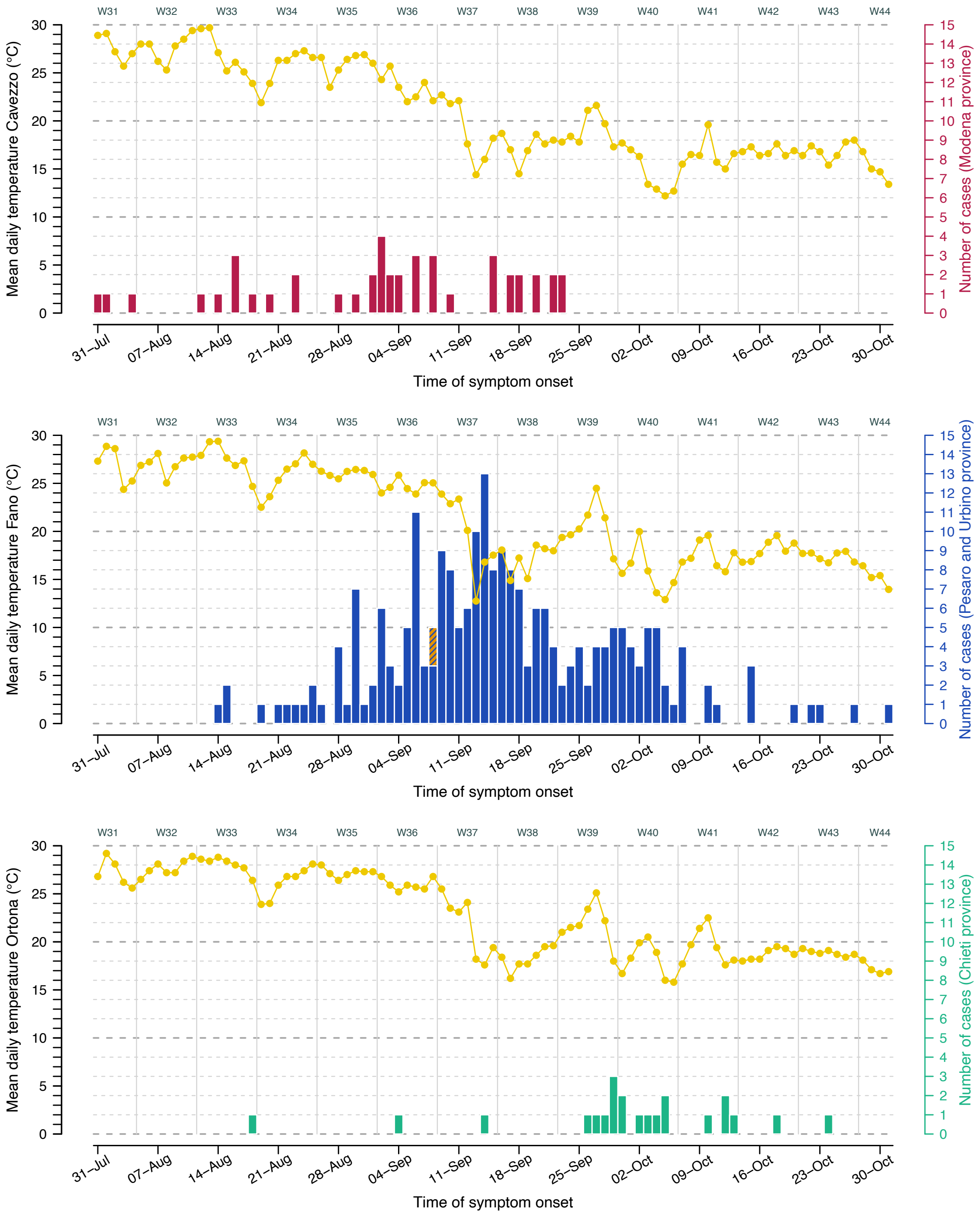

Figure S4. *Mean daily temperature (yellow) in the main affected towns and time series by date of symptom onset of the three principal foci: in the provinces of Modena (red), Pesaro and Urbino (blue), and Chieti (green). The two cases from the town in the province of Florence that spent a week in Fano while the outbreak in the region was ongoing are marked in orange with dashed blue lines. Week numbers, at the top, according to the ISO date standard.*

6. Age / sex distribution of the cases

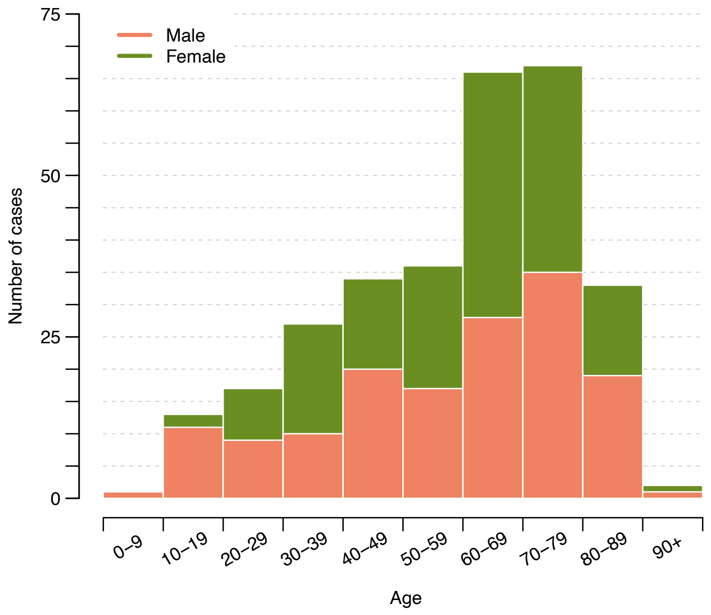

Figure S5. *Age distribution of the identified dengue cases. In orange male individuals, in green female.*

6.1. Age distribution of DENV-2 cases relative to the underlying population in Fano

To assess whether the observed age distribution of cases could be explained solely by the underlying population structure (see Figure S6), we performed a simulation analysis using the age distribution of residents in the municipality of Fano (4), where 75% of the cases included in this study were identified. We repeatedly sampled—10,000 times—the same number of DENV-2 cases observed in Fano (222 cases) from the population age distribution, assuming equal exposure to infection across age groups. The resulting simulated distributions were then compared with the observed age distribution of reported dengue cases (see Figure S6, panel C). The simulations showed that the proportion of cases aged ≥60 years in the observed data was consistently higher than expected under the assumption of age-independent exposure, indicating an overrepresentation of older adults among detected cases. Conversely, younger individuals were often underrepresented among cases relative to their proportion in the underlying population.

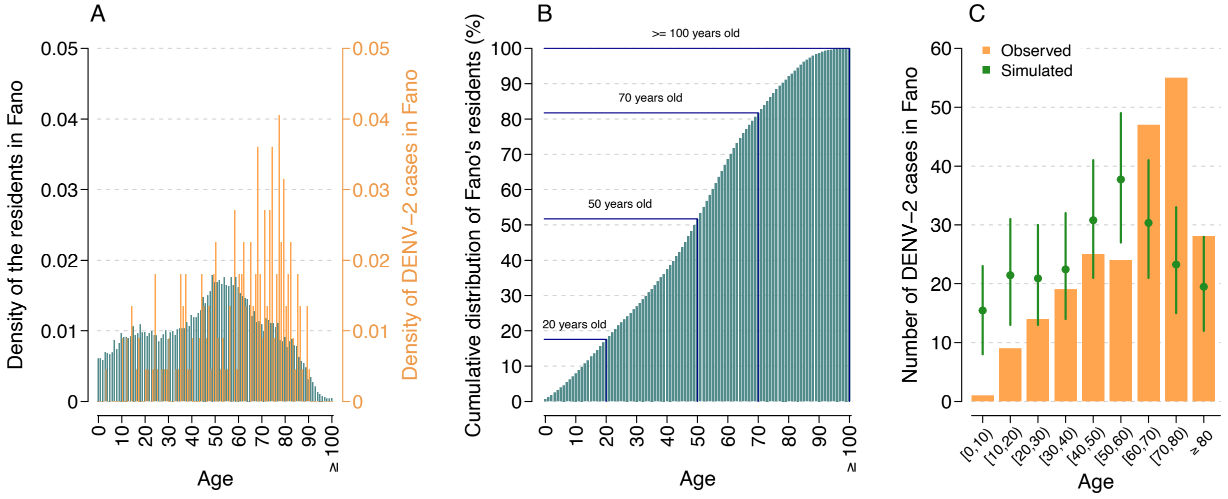

Figure S6. *Panel A shows the density function of the population of Fano by age (blue) and of DENV-2 cases identified in Fano (orange). Panel B shows the cumulative age distribution of the population of Fano. Panel C shows the observed number of cases by age group in the Municipality of Fano (orange) compared with simulated case counts under the assumption of equal exposure to infection across age groups (green). Green points indicate mean estimates; vertical lines represent the intervals from the 2.5th to the 97.5th percentiles across 10,000 simulations.*

7. Transmission chains reconstruction model

We developed a Bayesian spatio-temporal transmission model following a similar approach to what presented in previous studies (3,5,6). The model considers the force of infection over time exerted on an individual j, $\lambda_j(t)$, which accounts for the contribution of previously infected cases in the household (H), in the town (T), and for the overall circulation of the infection in the province (P). The force of infection can be defined as

$$\lambda_j(t) = \lambda_j^H(t) + \lambda_j^T(t) + \lambda_j^P(t)\,,$$

with

$$\lambda_j^H(t) = \sum_{i \in H_j} \lambda_{j,i}^H(t) = \sum_{i \in H_j} \alpha_{P_j}\, \Gamma(t - \tau_i\,;\, \mu,\, \sigma^2)\,,$$

$$\lambda_j^T(t) = \sum_{i \notin H_j\, \&\, i \in T_j} \lambda_{j,i}^T(t) = \sum_{i \notin H_j\, \&\, i \in T_j} \beta_{P_j}\, \eta e^{-\eta\, d_{i,j}}\, \Gamma(t - \tau_i\,;\, \mu,\, \sigma^2)\,,$$

$$\lambda_j^P(t) = \sum_{i \in P_j} \lambda_{j,i}^P(t) = \sum_{i \in P_j} \gamma_{P_j}\, \Gamma(t - \tau_i\,;\, \mu,\, \sigma^2)\,,$$

where H_j, T_j and P_j refer to the household, town and province of individual j, respectively, and α_{P_j}, β_{P_j}, and γ_{P_j} are province-specific free parameters scaling the transmissibility within these settings; $\eta e^{-\eta\, d_{i,j}}$ is the exponentially distributed distance kernel accounting for the probability that a transmission event occurs at a distance $d_{i,j}$ between the locations of likely exposure of individuals i and j; $\Gamma(t\,;\, \mu,\, \sigma^2)$ represents the distribution of the generation time at time t after infection, which we assumed to follow a Gamma distribution with parameters mean μ and variance σ^2; and τ_i is the unobserved time of infection of a potential infector i. The household force of infection $\lambda_j^H(t)$ considers all cases residing in the same house. The force of infection $\lambda_j^T(t)$ accounts for all potential infectors residing in the same town who are not cohabitants, incorporating the distance between the exposure locations of cases. The force of infection $\lambda_j^P(t)$ is assumed to be proportional to the overall number of cases observed in the province and aims to capture infection episodes that may have occurred in locations different from those reported during the outbreak investigations or involving unobserved cases. The analysis explicitly considered the duration of stay when accounting for the contribution of individuals who visited multiple affected towns or provinces, as for cases of the town in the province of Florence visiting Fano.

While setting-specific transmissibility parameters were estimated for the provinces of Modena and Pesaro and Urbino, the household, town, and province-level transmissibility parameters for Chieti were not directly estimated due to the small number of cases. Instead, they were assumed to be proportional to the average parameters from Modena and Pesaro and Urbino, scaled by a factor x that was jointly estimated during the calibration process:

$$\alpha_{Chieti} = x \cdot (\alpha_{Modena} + \alpha_{Pesaro\ and\ Urbino}) \,/\, 2\,,$$

$$\beta_{Chieti} = x \cdot (\beta_{Modena} + \beta_{Pesaro\ and\ Urbino}) \,/\, 2\,,$$

$$\gamma_{Chieti} = x \cdot (\gamma_{Modena} + \gamma_{Pesaro\ and\ Urbino}) \,/\, 2\,.$$

For the province of Florence, where the only three identified cases belonged to the same household and had all spent time in Fano (in the province of Pesaro and Urbino), only the household transmission parameter was considered and assumed to be equal to that estimated for the province of Pesaro and Urbino.

The first two symptomatic cases in Modena, the first three symptomatic cases in Pesaro and Urbino, and the first symptomatic case in Chieti, were assumed initial cases with unknown infector. We did not analyze potential sources of infection of individuals for whom a plausible site of exposure could not be determined during outbreak investigations. However, their contribution to the provincial force of infection was considered.

Given a set of model parameters, all plausible infectors for each case were considered. Among these, the individual k_j who contributed the most to the force of infection on individual j was identified as the most likely source of infection. The infection setting—household, town, or an unknown location within the province—was then determined as the one associated with the highest force of infection exerted by k_j on the case j. When the source of infection resulted to be either the household or the town, the individual k_j was considered as the most likely infector of case j. Otherwise, we assumed that the infection was acquired through an unobserved exposure event occurred within the province. The transmission distance between a case j and its infector k_j was computed as the geographical distance between the locations of likely exposure of the two individuals.

We assumed that the incubation period for dengue in humans—defined as the time interval between infection and symptom onset—lasts between 3 and 10 days (3,7,8). In our study, free model parameters are: $[\alpha, \beta, \gamma]_{Pesaro\ and\ Urbino}$, $[\alpha, \beta, \gamma]_{Modena}$, x, η, μ, and σ^2. The infection times τ_i of the identified cases are considered unobserved events. Free model parameters and unobserved events were estimated using a Markov Chain Monte Carlo (MCMC) approach, with likelihood function

$$L = \prod_j \lambda_j(\tau_j)\, e^{-\sum_{t<\tau_j} \lambda_j(t)} \ .$$

Exploration of free model parameters was conducted using the Metropolis-Hasting algorithm. Additionally, at each MCMC iteration, 20% of all infection times τ_i were randomly sampled to explore uncertainty in the unobserved time of infection of observed cases. A total of 2,000,000 MCMC iterations, with a burn-in period of 200,000 iterations, were performed.

The epidemiological results presented in the paper are based on 50,000 sets of model parameters and infection times, randomly sampled from their joint posterior distribution obtained via the MCMC approach.

7.1. Generation time estimates

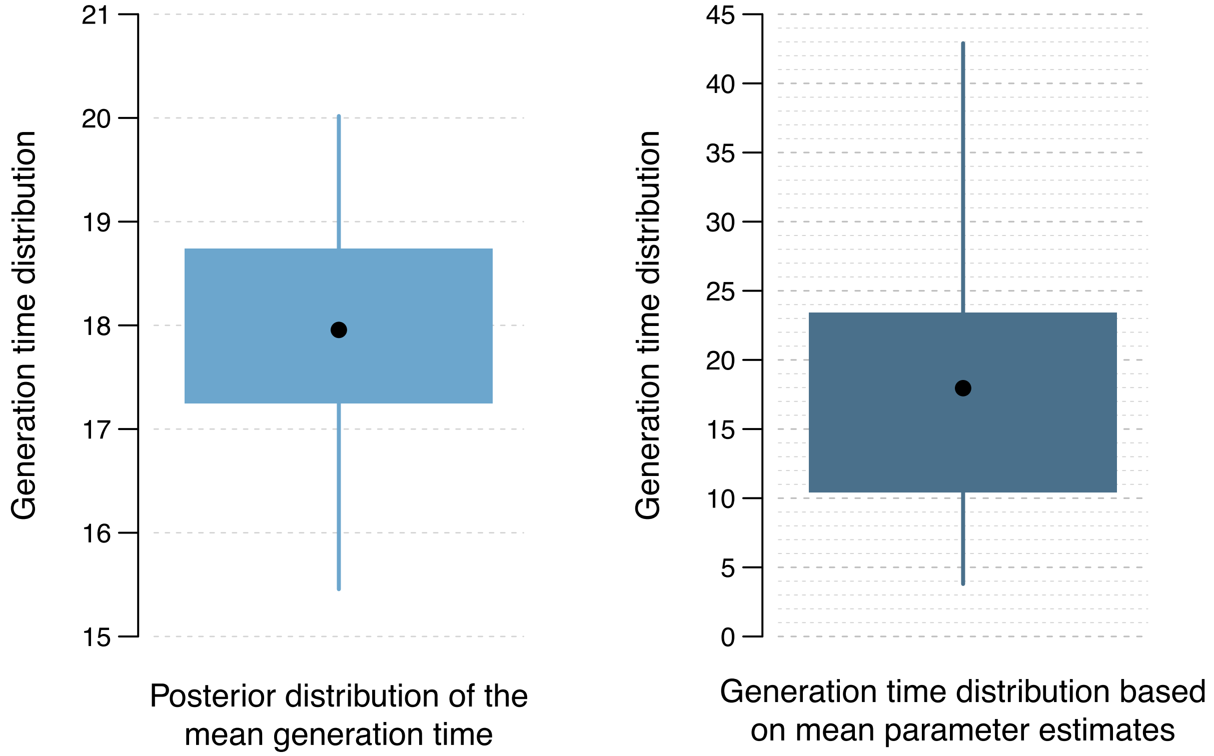

Figure S7. *Estimated generation time distribution for dengue. On the left, posterior distribution of the mean generation time; on the right, the generation time distribution based on the mean parameter estimates (i.e., gamma distribution with mean = 18.0 and standard deviation = 10.3). Points: mean, box: 50% CrI, lines: 95% CrI.*

7.2. Map of the transmission chain reconstruction in Fano center

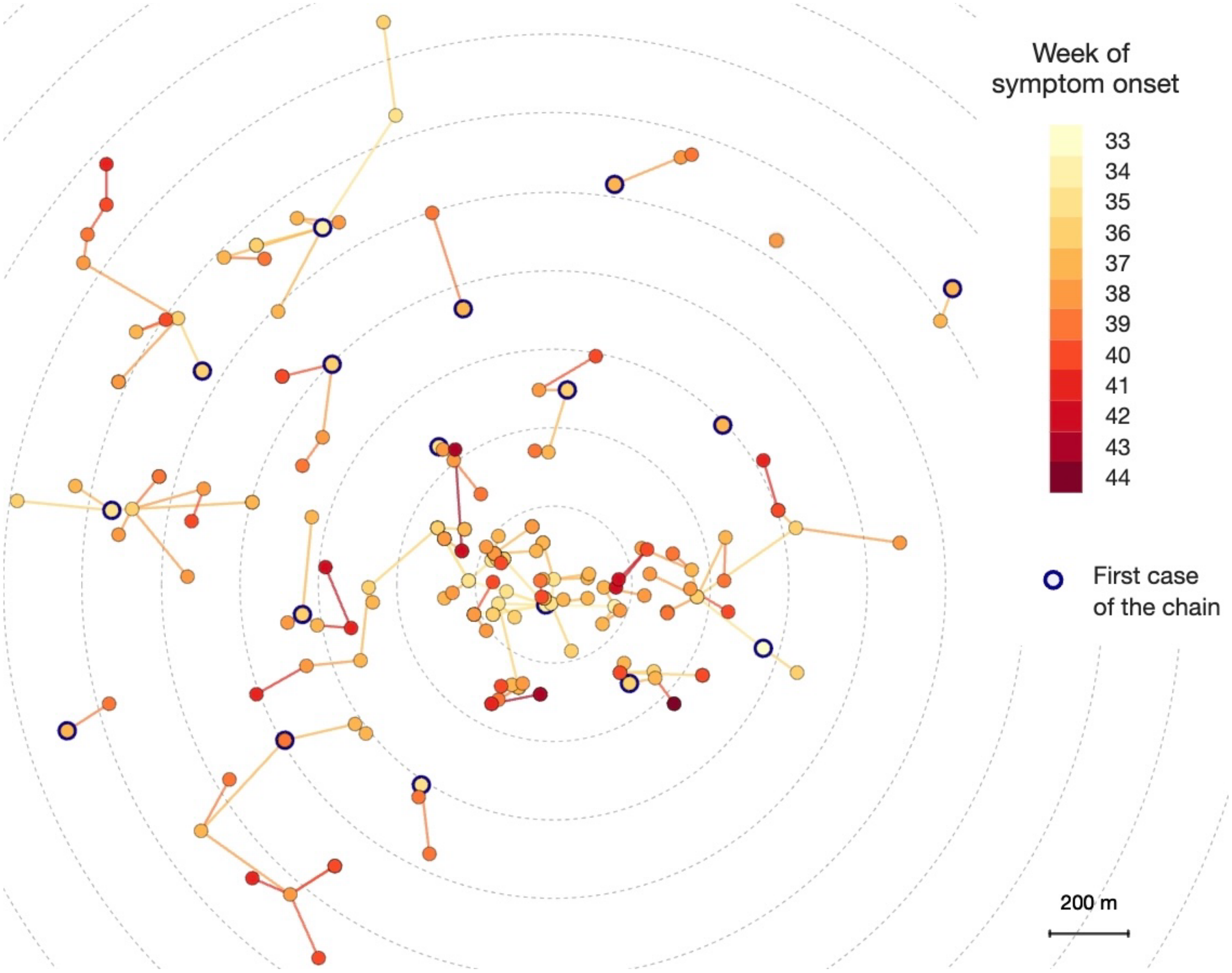

Figure S8. *Consensus transmission chains identified in the epicenter of the outbreak in Fano town. The colors of the cases (circles) indicate their week of symptom onset, and the color of the transmission link refers to the time of symptom onset of the infectee. The blue border of some cases indicate they are the first case of a transmission chain. The grey concentric circles indicate intervals of 200 meters from the centroid of the geolocated cases with symptom onset on weeks 33 and 34 according to the ISO date standard.*

8. Net reproduction number

To estimate the net reproduction number $R(t)$ over time in each of the affected provinces, we used a similar approach to (3,9). We assumed that the daily number of identified positive cases can be approximated by a Poisson distribution as

$$C(t) \sim Pois\left(R(t)\sum_{s=0}^{t}\Gamma(s)\,C(t-s)\right),$$

where

- $C(t)$ is the daily number of identified infections (from $t = 1$ to $t = T$);
- $R(t)$ is the net reproduction number at time t;
- $\Gamma(s)$ is the generation time distribution of dengue at time s, as estimated by transmission chain reconstruction model.

The likelihood L of the observed time-series of cases from day 1 to day T is thus given by

$$L = \prod_{t=1}^{T} P\left(C(t);\ R(t)\sum_{s=1}^{t}\Gamma(s)\,C(t-s)\right),$$

where $P(k;\lambda)$ is the probability mass function of a Poisson distribution (i.e., the probability of observing k events if these events occur at rate λ). The posterior distribution of the net reproduction number $R(t)$ at each $t \in [1, T]$ is estimated by using an MCMC Metropolis-Hastings's algorithm.

The first two cases in the town in the province of Florence, who were exposed to the infection in Fano, are explicitly accounted for in the model by considering them solely as secondary cases and not as potential primary cases within that location. The resulting daily $R(t)$ estimates are averaged over a centered window of two weeks.

9. Regression analysis

We investigated potential determinants of the number of secondary infections generated by each case according to the reconstructed consensus chain. To do this, we considered the following explanatory variables characterizing each case:

a) age,
b) sex,
c) population density in their location of likely exposure,
d) normalized difference vegetation index (NDVI) in their location of likely exposure,
e) a dichotomous variable (pre-post) identifying their timing of symptom onset relative to outbreak detection,
f) the mean temperature in town from the time of infection of the individual and for 18 days (~ one generation time),
g) a binary variable indicating whether vector control interventions were already implemented in their location of exposure prior to their symptom onset,
h) and the reporting delay of the individual.

Graphical exploratory techniques, Cook's Distance, and Variance Inflation Factor (VIF) were used to check for outliers and potential collinearity. Visual inspection of model residuals was carried out to assess the integrity of statistical assumptions underlying the considered regression models.

Exploratory analyses showed two outliers which were excluded from the regression model, which were later confirmed as influential points in the regression analyses by having Cook's distance > $\sqrt{k/n}$, with k the number of independent variables, and n the number of data points. Moreover, variables e, f, g, and h were highly collinear (see Figure S9). In particular, for variable g, control interventions occurred only post-outbreak detection; no case with symptom onset before outbreak detection had a control intervention implemented in their likely site of exposure before. We thus decided to proceed with two complementary models: one retaining variable e (pre-post: identifying their timing of symptom onset relative to outbreak detection) that summarizes all others (f, g, h) – model 1 –; and a second model that considers f, g, h instead of e – model 2 –.

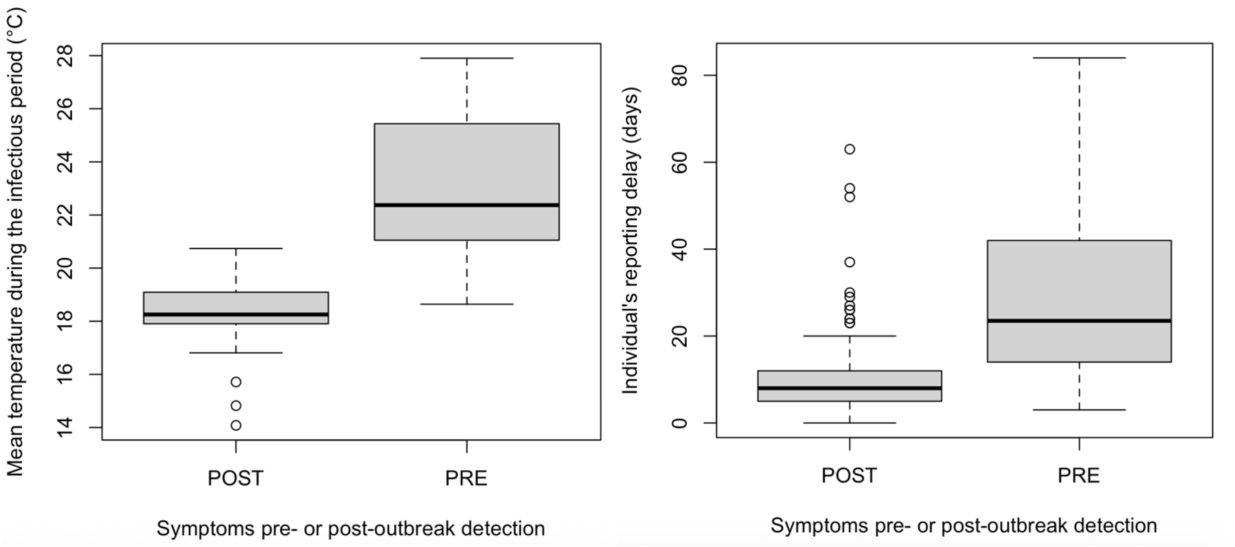

Figure S9. *Boxplots of the temperature (left) and reporting delay (right) distributions while comparing those cases with symptom onset pre- or post-outbreak detection.*

We fitted a generalized linear model (NB-GLM) with log link to investigate the risk factors associated with the transmission of the virus. We assumed a negative binomial distribution for the dependent variable, represented by the number of cases generated by each infected individual (y_i). For each potential infector, we considered as independent variables their age, sex, population density and NDVI in their location of likely exposure, and a dichotomous variable (pre-post) identifying their timing of symptom onset relative to outbreak detection. The adopted model equation is

$$y_i \sim NegBin(\mu_i, \theta) \ ,$$

$$log(\mu_i) = \alpha + \alpha_1 \cdot age_i + \alpha_2 \cdot sex_i + \alpha_3 \cdot p.dns_i \ + \alpha_4 \cdot ndvi_i +$$

$$+ \alpha_5 \cdot pre.post_i \ ,$$

where the subscript i refers to the specific individual case; y_i is the number of secondary infections generated by individual i according to the consensus transmission chain; μ_i is the expected number of secondary cases; θ is the overdispersion parameter of the negative binomial distribution; α is the intercept; and α_X are the slope coefficients for each of the independent variables. Estimated parameters of this model are in Table S1.

Table S1. *Regression results of the GLM considering the pre-post factor (model 1).*

Characteristic	IRR[1]	95% CI[1]	p-value
age	1.00389	0.99634, 1.01167	0.3180325758
sex			
F	—	—	
M	1.41274	1.04431, 1.91710	0.0253816021
human_density	1.00272	0.99614, 1.00930	0.4043110190
ndvi	0.46872	0.03556, 5.70034	0.5543302383
pre_post			
POST	—	—	
PRE	3.72588	2.71222, 5.17190	0.0000000000

[1] IRR = Incidence Rate Ratio, CI = Confidence Interval

Log-likelihood = -307; AIC = 627; BIC = 652; Residual df = 262

No collinearity was detected by computing the variance inflation factor (VIF) (VIF $\leq$ 1.08 for all independent variables). Visual inspection of model residuals did not indicate any violation of the model's assumptions.

We fitted a second regression model aiming to detail the contribution of pre- and post-outbreak detection by considering the following three variables instead of the dichotomous pre-post variable: the mean temperature in town from the time of infection of the individual and for 18 days, a binary variable indicating whether vector control interventions were already implemented in their location of exposure prior to their symptom onset, and the reporting delay of the individual. In this case, the model is:

$$y_i \sim NegBin(\mu_i, \theta)\,,$$

$$log(\mu_i) = \alpha + \alpha_1 \cdot age_i + \alpha_2 \cdot sex_i + \alpha_3 \cdot p.dns_i + \alpha_4 \cdot ndvi_i +$$

$$+ \alpha_5 \cdot temperature_i + \alpha_6 \cdot control_i + \alpha_7 \cdot rep.delay_i.$$

Estimated parameters of this model are in Table S2.

Table S2. Regression results of the GLM considering, instead of the pre-post factor, the temperature, the presence of control measures, and the reporting delay (model 2).

Characteristic	IRR[1]	95% CI[1]	p-value
age	1.00091	0.99339, 1.00867	0.8155422031
sex			
F	—	—	
M	1.30148	0.97736, 1.73838	0.0719233994
human_density	1.00211	0.99604, 1.00812	0.4864868744
ndvi	0.13835	0.01176, 1.47458	0.1072825056
report_delay	1.00431	0.99358, 1.01492	0.4266452846
control_sym			
NO	—	—	
YES	0.59011	0.36692, 0.93403	0.0264350189
temperature_mgt	1.19766	1.11320, 1.28960	0.0000011500

[1] IRR = Incidence Rate Ratio, CI = Confidence Interval

Log-likelihood = -292; AIC = 601; BIC = 633; Residual df = 260

Visual inspection of model residuals did not indicate any violation of the model's assumptions.

To account for potential transmission heterogeneities between the three main foci analyzed, the above regressions were repeated by considering the province of the cases as either a fixed or random effect. Results obtained in the sensitivity analysis were consistent with those obtained in our baseline analysis (see Tables S3, S4, S5, and S6).

Table S3. Regression results of the GLM with the pre-post factor and the provinces as an additional factor (model 1 with the provinces as fixed effects).

Characteristic	IRR[1]	95% CI[1]	p-value
age	1.00459	0.99702, 1.01239	0.2417135183
sex			
F	—	—	
M	1.38054	1.02169, 1.87097	0.0357550249
human_density	1.00149	0.99487, 1.00811	0.6500633163
ndvi	1.08361	0.06780, 16.1935	0.9538550915
pre_post			
POST	—	—	
PRE	3.96577	2.86760, 5.54115	0.0000000000
factor(province)			
CHIETI	—	—	
MODENA	0.60365	0.24762, 1.64147	0.2759851405
PESARO-URBINO	0.91403	0.41083, 2.33186	0.8313455294

[1] IRR = Incidence Rate Ratio, CI = Confidence Interval

Log-likelihood = -305; AIC = 628; BIC = 660; Residual df = 260

Table S4. Regression results of the GLMM with the pre-post factor and the provinces as random effects (model 1 with the provinces as random effects).

Characteristic	exp(Beta)	95% CI[1]	p-value
age	1.00389	0.99625, 1.01159	0.3187874017
sex			
F	—	—	
M	1.41274	1.04265, 1.91419	0.0257827308
human_density	1.00272	0.99617, 1.00932	0.4165682068
ndvi	0.46872	0.03711, 5.91941	0.5581210413
pre_post			
POST	—	—	
PRE	3.72588	2.69917, 5.14313	0.0000000000
province.sd__(Intercept)	0.00003	1.47457, 8.82206	

[1] CI = Confidence Interval

Log-likelihood = -307; AIC = 629; BIC = 658; Residual df = 260

Table S5. Regression results of the GLM considering, instead of the pre-post factor, the reporting delay, the presence of control measures, and the temperature, with the provinces as an additional factor (model 2 with the provinces as fixed effects).

**Characteristic**	**IRR**[1]	**95% CI**[1]	**p-value**
age	1.00147	0.99411, 1.00909	0.7017141248
sex			
F	—	—	
M	1.28546	0.97322, 1.70355	0.0778157488
human_density	1.00055	0.99462, 1.00643	0.8549885825
ndvi	0.47029	0.03625, 5.54974	0.5568512878
report_delay	1.00260	0.99235, 1.01274	0.6156618144
control_sym			
NO	—	—	
YES	0.56515	0.35282, 0.89074	0.0154214093
temperature_mgt	1.21563	1.13161, 1.30677	0.0000000956
factor(province)			
CHIETI	—	—	
MODENA	0.77857	0.34198, 2.01888	0.5694093926
PESARO-URBINO	1.35551	0.65169, 3.31111	0.4489177359

[1] IRR = Incidence Rate Ratio, CI = Confidence Interval

Log-likelihood = -288; AIC = 599; BIC = 638; Residual df = 258

Table S6. *Regression results of the GLMM considering, instead of the pre-post factor, the reporting delay, the presence of control measures, and the temperature, with the provinces as random effects (model 2 with the provinces as random effects).*

Characteristic	exp(Beta)	95% CI[1]	p-value
age	1.00126	0.99374, 1.00883	0.7441587853
sex			
F	—	—	
M	1.29075	0.97110, 1.71561	0.0787544564
human_density	1.00116	0.99507, 1.00728	0.7101634342
ndvi	0.28404	0.02012, 4.00902	0.3513936968
report_delay	1.00331	0.99292, 1.01381	0.5339943645
control_sym			
NO	—	—	
YES	0.57370	0.36044, 0.91313	0.0191202886
temperature_mgt	1.20796	1.12227, 1.30020	0.0000004844
province.sd__(Intercept)	0.18405	1.41096, 87.8071	

[1] CI = Confidence Interval

Log-likelihood = -291; AIC = 602; BIC = 638; Residual df = 258

All regression analyses were performed using R (R Project for Statistical Computing, software version 4.4.2), including libraries *MASS*, *car*, *performance*, *glmmTMB*, and *gtsummary*.

References

1. Italian Ministry of Health. National Arbovirus Plan (Piano Nazionale di prevenzione, sorveglianza e risposta alle Arbovirosi) 2020–2025. [Internet]. 2019. Available from: https://www.salute.gov.it/imgs/C_17_pubblicazioni_2947_allegato.pdf

2. European Commission. Commission Implementing Decision (EU) 2018/945 of 22 June 2018 on the communicable diseases and related special health issues to be covered by epidemiological surveillance as well as relevant case definitions. OJ L [Internet]. 2018 Jun 22. Available from: http://data.europa.eu/eli/dec_impl/2018/945/oj

3. Rovida F, Faccini M, Molina Granè C, Cassaniti I, Senatore S, Rossetti E, et al. The 2023 dengue outbreak in Lombardy, Italy: A one-health perspective. Travel Med Infect Dis. 2025 Mar 1;64:102795. doi:10.1016/j.tmaid.2025.102795

4. ISTAT. All municipalities by age [Internet]. [cited 2026 Mar 18]. Available from: https://esploradati.istat.it/databrowser/#/en/dw/categories/IT1,POP,1.0/POP_POPULATION/DCIS_POPRES1/IT1,22_289_DF_DCIS_POPRES1_24,1.0

5. Guzzetta G, Vairo F, Mammone A, Lanini S, Poletti P, Manica M, et al. Spatial modes for transmission of chikungunya virus during a large chikungunya outbreak in Italy: a modeling analysis. BMC Med. 2020 Aug 7;18(1):1. doi:10.1186/s12916-020-01674-y

6. Salje H, Lessler J, Paul KK, Azman AS, Rahman MW, Rahman M, et al. How social structures, space, and behaviors shape the spread of infectious diseases using chikungunya as a case study. Proc Natl Acad Sci. 2016 Nov 22;113(47):13420–5. doi:10.1073/pnas.1611391113

7. Guzzetta G, Marques-Toledo CA, Rosà R, Teixeira M, Merler S. Quantifying the spatial spread of dengue in a non-endemic Brazilian metropolis via transmission chain reconstruction. Nat Commun. 2018 Jul 19;9(1):1. doi:10.1038/s41467-018-05230-4

8. Chan M, Johansson MA. The Incubation Periods of Dengue Viruses. PLOS ONE. 2012 Nov 30;7(11):e50972. doi:10.1371/journal.pone.0050972

9. Cori A, Ferguson NM, Fraser C, Cauchemez S. A New Framework and Software to Estimate Time-Varying Reproduction Numbers During Epidemics. Am J Epidemiol. 2013 Nov 1;178(9):9. doi:10.1093/aje/kwt133
